# Establishing seasonal and alert influenza thresholds in Cambodia using the WHO method: implications for effective utilization of influenza surveillance in the tropics and subtropics

**DOI:** 10.5365/WPSAR.2017.8.1.002

**Published:** 2017-03-15

**Authors:** Sovann Ly, Takeshi Arashiro, Vanra Ieng, Reiko Tsuyuoka, Amy Parry, Paul Horwood, Seng Heng, Sarah Hamid, Katelijn Vandemaele, Savuth Chin, Borann Sar, Yuzo Arima

**Affiliations:** a Communicable Disease Control Department, Ministry of Health, Phnom Penh, Cambodia.; b WHO Representative Office in Cambodia, Phnom Penh, Cambodia.; c Infectious Disease Surveillance Center, National Institute of Infectious Diseases, Tokyo, Japan.; d School of Medicine, Tokyo Medical and Dental University, Tokyo, Japan.; e Virology Unit, Institut Pasteur in Cambodia, Phnom Penh, Cambodia.; f Emerging Disease Surveillance and Response, World Health Organization Regional Office for the Western Pacific, Manila, Philippines.; g Global Influenza Programme, World Health Organization, Geneva, Switzerland.; h National Public Health Laboratory, National Institution of Public Health, Phnom Penh, Cambodia.; i Influenza Program, United States Centers for Disease Control and Prevention, Phnom Penh, Cambodia.

## Abstract

**Objective:**

To establish seasonal and alert thresholds and transmission intensity categories for influenza to provide timely triggers for preventive measures or upscaling control measures in Cambodia.

**Methods:**

Using Cambodia’s influenza-like illness (ILI) and laboratory-confirmed influenza surveillance data from 2009 to 2015, three parameters were assessed to monitor influenza activity: the proportion of ILI patients among all outpatients, proportion of ILI samples positive for influenza and the product of the two. With these parameters, four threshold levels (seasonal, moderate, high and alert) were established and transmission intensity was categorized based on a World Health Organization alignment method. Parameters were compared against their respective thresholds.

**Results:**

Distinct seasonality was observed using the two parameters that incorporated laboratory data. Thresholds established using the composite parameter, combining syndromic and laboratory data, had the least number of false alarms in declaring season onset and were most useful in monitoring intensity. Unlike in temperate regions, the syndromic parameter was less useful in monitoring influenza activity or for setting thresholds.

**Conclusion:**

Influenza thresholds based on appropriate parameters have the potential to provide timely triggers for public health measures in a tropical country where monitoring and assessing influenza activity has been challenging. Based on these findings, the Ministry of Health plans to raise general awareness regarding influenza among the medical community and the general public. Our findings have important implications for countries in the tropics/subtropics and in resource-limited settings, and categorized transmission intensity can be used to assess severity of potential pandemic influenza as well as seasonal influenza.

## Introduction

Influenza poses a substantial health and economic burden with high morbidity and mortality in temperate regions. (*1*-*4*) The burden of influenza in the tropics and subtropics is not well understood, although growing evidence suggests that it is comparable to that of temperate regions. (*3*-*7*) Furthermore, while yearly variations may occur, seasonality also appears to exist in most tropical and subtropical regions. (*8*-*11*) Therefore, it is essential to analyse influenza surveillance data in a practical and efficient manner to inform decision-making regarding influenza in the tropics and subtropics.

Recently, based on sentinel surveillance data from Cambodia’s National Influenza Center established in 2006, distinct seasonality for influenza in Cambodia was demonstrated. (*12*, *13*) Using the proportion of influenza-like illness (ILI) patient samples positive for influenza, the influenza season appeared to be between June and December, coinciding with the rainy season. However, such findings have not yet been fully used for routine public health practice. Establishing specific influenza thresholds at the national level for season onset and intensity levels could provide timely triggers for public health measures, such as awareness-raising for prevention, upscaling control measures and resource allocation. Various methods such as visual inspection, pre-set constant values and the moving epidemic method have been implemented in countries to signal season onset. (*8*, *14*-*16*)

In the present study, a simple method proposed by the World Health Organization (WHO) was used to establish seasonal and alert influenza thresholds for Cambodia to better inform public health decision-making regarding influenza. (*17*) The WHO method allows for monitoring intensity of not only seasonal influenza but also potential pandemics. A key lesson learnt from the 2009 pandemic was that WHO and most countries were not sufficiently prepared to assess the severity of a mild pandemic to inform timely risk management and communications. Following the International Health Regulations review committee recommendations, WHO is developing the Pandemic Influenza Severity Assessment (PISA) framework. (*18*) To assess severity of a pandemic, comparison with historical data is important. Establishing influenza alert thresholds allows for the comparison of data during a pandemic relative to historical seasonal data. To our knowledge, this is one of the first documented assessments and applications of the WHO method for threshold setting in the tropics or subtropics.

## Methods

### Influenza surveillance system in Cambodia

Cambodia’s influenza surveillance system has two key components: (1) weekly syndromic ILI surveillance; and (2) laboratory testing of specimens collected from ILI patients for influenza virus, both of which come from sentinel sites. While there is also a surveillance system for severe acute respiratory illness (SARI), it was not included because only the past three years’ data were available with too few SARI cases to establish thresholds.

There were eight sentinel sites in operation during the study period of week 1 of 2009 to week 25 of 2015, including four health centres (HCs) in Battambang, Kampong Cham, Kampot and Mundol Kiri provinces; two paediatric hospitals in Phnom Penh and Siem Reap provinces; and two general hospitals in Svay Rieng and Takeo provinces. Not all sentinel sites provided data during the entire study period. The following contributed data during shorter periods: HCs in Kampot and Mundol Kiri provinces (since 2010), Svay Rieng Referral Hospital (since mid-2009) and Takeo Provincial Hospital (2009–2012). Thus, there were six to eight sentinel sites contributing data at a given time.

An ILI case was defined as a person presenting with sudden onset of fever (temperature > 38 °C) and cough and/or sore throat in the absence of other diagnosis. Although the number of samples collected varied yearly due to minor protocol changes, approximately 5–10 nasopharyngeal swabs per site per week were collected from ILI patients. Collected specimens were laboratory tested for influenza virus at the National Institute of Public Health and/or Institut Pasteur in Cambodia, except for the site in Battambang province where the testing facility is in the province. Viral RNA was extracted using commercial extraction kits and amplified with reverse transcription polymerase chain reaction (RT–PCR) using standard protocols. (*12*, *13*)

### Data sources and parameters to monitor influenza activity

The following data were extracted from the sentinel surveillance system from four data sources: number of (1) new outpatients, (2) ILI patients, (3) specimens collected among ILI patients for laboratory testing, and (4) influenza positives among specimens collected. To establish thresholds, data from week 18 of 2010 to week 17 of 2014 were used, totalling 867 266 outpatients, 36 885 ILI patients, 9136 laboratory specimens from ILI patients and 1482 laboratory-confirmed influenza cases. Data before week 18 of 2010 were not used for threshold setting due to the 2009 pandemic. Three parameters were calculated for each week: (1) proportion of ILI patients among all outpatients (proportion ILI); (2) proportion of laboratory specimens from ILI patients positive for influenza (proportion positive); and (3) an ILI-influenza composite variable (composite), the product of proportion ILI and proportion positive proposed by Tay et al. (*19*)

### Establishing seasonal and alert thresholds and categorizing transmission intensity

**Fig. 1:**
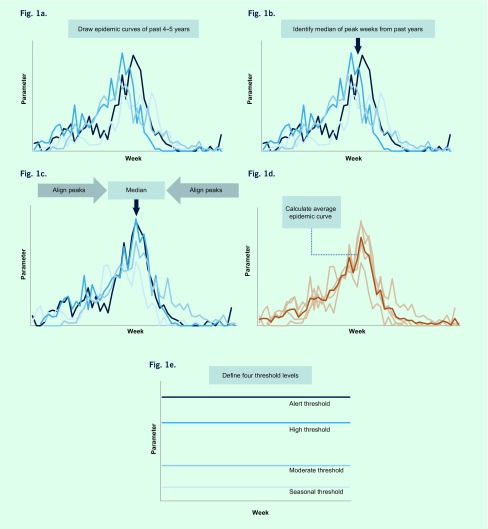
Illustration of the WHO method to establish four levels of thresholds (adapted from WHO Global Epidemiological Surveillance Standards for Influenza) based on proportion of laboratory specimens from ILI patients positive for influenza (proportion positive) data from Cambodia, 2009 to 2015)

We adapted the WHO method described in the WHO Global Epidemiological Surveillance Standards for Influenza (WHO manual) to establish seasonal and alert thresholds for the three parameters described above, with some modifications (**Fig. 1**). (*17*)

A. Draw epidemic curves for previous 4 or 5 years with calendar week on *x*-axis and parameter on *y*-axis.

B. Identify median of peak weeks from past years.

C. Shift the data from previous years to the point where their respective peaks align on median of peak weeks identified in B.

D. Calculate an average over the years for each week to create the average epidemic curve.

E. Define thresholds levels.

First, to define different thresholds we drew weekly epidemic curves for the past 4–5 years (**Fig. 1a**). Next, the median week of peak occurrence was identified from these years (**Fig. 1b**). Then, respective peaks from previous years were aligned on the median week (**Fig. 1c**). An average epidemic curve, which captures a typical influenza season’s temporal distribution and amplitude, was drawn by calculating an arithmetic mean over the years for each week (**Fig. 1d**).

Finally, four threshold levels were determined: (1) seasonal, (2) moderate, (3) high, and (4) alert (**Fig. 1e**). As the thresholds are context-specific, we explored a range of candidates, including those recommended in the WHO manual for seasonal and alert thresholds, (*17*) those proposed by Tay et al. (*19*) and those proposed through key stakeholder discussions. Final selections were based on consensus among national and international experts for technical and practical reasons based on several meetings with in-depth discussions. To define season onset (seasonal threshold), the median value of all weeks during the study period (*17*) was used since we assumed seasonality in influenza activity with approximately half of the year being in-season and the other half off-season. For the moderate threshold, which defines a mild season set between high and seasonal thresholds, we explored the mean and the median values of all weeks during the in-season weeks during the study period (i.e. all weeks above the seasonal threshold). For the high threshold, which defines a higher than average season, we compared the peak value of the average and median epidemic curves. (*17*, *19*) Alert threshold defines extraordinarily severe seasons such as pandemics, and the upper 95% and 90% confidence interval (CI) and the 95th and 90th percentile of the peak values were explored. (*17*, *19*) Based on these four threshold levels, intensity of influenza transmission was classified into five categories: (1) out of season, (2) low, (3) moderate, (4) high, and (5) extraordinary.

### Assessment of thresholds

Data for the three parameters from week 1 of 2009 to week 25 of 2015 were plotted against the established thresholds. Influenza season was defined to start when the parameter increased above the seasonal threshold and to end when the parameter declined below the threshold. The number of times per year the seasonal threshold was crossed was used to assess the validity of the seasonal threshold; assuming one influenza season per year in Cambodia based on historic data, (*10*, *12*, *13*) additional detected seasons were considered false alarms. We also compared results from two conventional rules to declare season onset: the first-week-declaration rule, where onset is declared on the first week the threshold is crossed and the two-consecutive-week-declaration rule, where onset is declared when the threshold is crossed for two consecutive weeks. (*19*) As additional sensitivity analysis, thresholds were re-calculated including 2009/2010 data to assess the degree of the pandemic season’s impact on the parameters and thresholds. The most recent data from week 18 of 2014 provided an opportunity to assess the proposed thresholds using data not included in establishing the thresholds, as would be the case in practice.

### Ethics statement

The ILI and influenza surveillance system is a public health activity organized by the Ministry of Health in Cambodia and has standing authorization from the National Ethics Committee, Cambodia. Data that could potentially identify individuals are not included.

## Results

### Comparison of parameters to monitor influenza activities

**Fig. 2:**
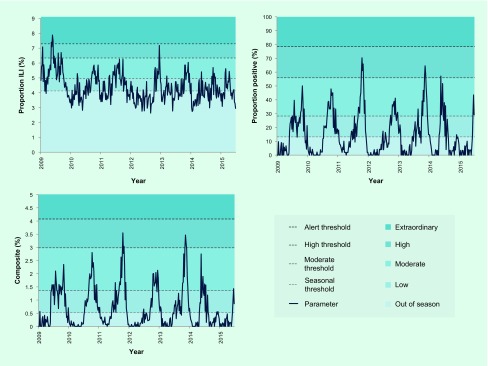
Surveillance data from 2009 to 2015 plotted against established thresholds and intensity categorization for three parameters: (1) proportion of ILI patients among all outpatients (proportion ILI), (2) proportion of laboratory specimens from ILI patients positive for influenza (proportion positive), and (3) the product of proportion ILI and proportion positive (composite)

When 2009–2015 data were plotted for the three parameters, proportion ILI showed extensive weekly fluctuations with no clear seasonal pattern, but proportion positive and composite both exhibited clear seasonality, peaking between October and December for the majority (5/6) of seasons (**Fig. 2**).

### Establishment and assessment of seasonal and alert thresholds and intensity categorization

Four threshold levels were established (**Table 1**), defining five categories of transmission intensity (**Fig. 2**). Each threshold was based on a different criterion process described below.

**Table 1:** Values of established thresholds for three parameters: (1) proportion of ILI patients among all outpatients (proportion ILI), (2) proportion of laboratory specimens from ILI patients positive for influenza (proportion positive), and (3) the product of proportion ILI and proportion positive (composite)

Threshold level	Proportion ILI (%)	Proportion positive (%)	Composite (%)
Seasonal	4.1	13.2	0.5
Moderate	5.0	28.2	1.4
High	6.3	56.0	3.0
Alert	7.3	78.6	4.1

#### Seasonal threshold

While visual inspection indicated one season per year for most years based on the proportion positive and composite parameters (**Fig. 2**), with the first-week-declaration rule, the seasonal threshold was crossed multiple times for most years (**Table 2**). While multiple-season years were observed for all parameters, the frequency was greatest for the proportion ILI and least for the composite. The two-consecutive-week-declaration rule reduced the frequency considerably regardless of parameter. Notably, all years were shown to have a single season using the composite.

**Table 2:** Number of seasons detected from 2009 to 2015 using the median value from all weeks as the seasonal threshold, with either the first-week-declaration rule or two-consecutive-week-declaration rule for the three parameters: proportion ILI, proportion positive and composite

Season*	Proportion ILI		Proportion positive		Composite	
	1^st^ week**	2^nd^ week***	1^st^ week**	2^nd^ week***	1^st^ week**	2^nd^ week***
2009/2010	5	2	3	1	2	1
2010/2011	7	3	1	1	1	1
2011/2012	6	2	4	1	1	1
2012/2013	7	2	3	3	3	1
2013/2014	9	4	6	1	6	1
2014/2015	10	5	3	1	4	1

* Starts on week 18 and ends on week 17 of the following year.

** Start or end of season declared on the first week threshold was crossed.

*** Start or end of season declared when threshold is crossed for two consecutive weeks.

#### Alert threshold

Exploring a range of CI and percentiles, the upper 90% CI of the average epidemic curve peak amplitude was adopted (**Fig. 2**) as suggested in the WHO manual (*17*) and used previously in an Australian study. (*19*) The upper 90% CI had consistently higher values than the 90th or 95th percentiles for proportion positive and composite (data not shown). The only time the alert threshold was crossed was during the 2009 pandemic year with proportion ILI (**Table 3**).

**Table 3:** Duration of each intensity category* in weeks for influenza seasons from 2009 to 2015 for the three parameters: proportion ILI, proportion positive and composite

Season**	Out of season (weeks)	Low (weeks)	Moderate (weeks)	High (weeks)	Extraordinary (weeks)
Proportion ILI					
2009/2010	21	9	15	4	3
2010/2011	25	15	12	0	0
2011/2012	21	17	14	0	0
2012/2013	36	8	7	1	0
2013/2014	29	16	7	0	0
2014/2015	27	16	9	0	0
Proportion positive					
2009/2010	26	18	8	0	0
2010/2011	26	14	12	0	0
2011/2012	28	11	9	4	0
2012/2013	29	13	10	0	0
2013/2014	27	15	7	3	0
2014/2015	38	7	6	1	0
Composite					
2009/2010	25	14	13	0	0
2010/2011	25	16	11	0	0
2011/2012	25	15	10	2	0
2012/2013	33	10	9	0	0
2013/2014	28	14	8	2	0
2014/2015	38	9	5	0	0

* Intensity category is raised/lowered on the first week each threshold is crossed except for the seasonal threshold in which intensity is raised/lowered when the threshold is crossed for two consecutive weeks.

** Starts on week 18 and ends on week 17 of the following year.

#### High threshold

Peak amplitude of the average epidemic curve was adopted (**Fig. 2**) because average and median epidemic curves were found to be similar and sensitivity analysis including or excluding 2009/2010 data suggested that the average epidemic curve was more stable than the median when number of years used for threshold establishment was few (data not shown). Based on the proportion positive and composite, the 2009 pandemic year did not reach the high threshold (**Table 3**).

#### Moderate threshold

The mean rather than the median value was selected as it would better distribute the in-season weeks between the low and moderate levels (**Fig. 2**). Given the distribution of the data during the in-season period, the latter would set the threshold at a considerably low level, close to the seasonal threshold, and make the moderate threshold practically less useful.

### Sensitivity analysis of thresholds with 2009 pandemic year data and application of thresholds to 2014–2015 data

**Fig. 3:**
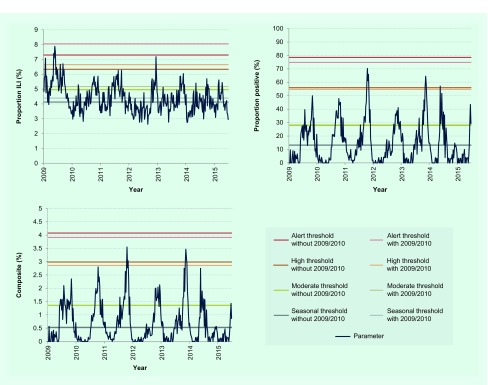
Sensitivity analysis of established thresholds for the three parameters including or excluding 2009 pandemic year data

Finally, we tested whether inclusion of data from the 2009 pandemic for threshold determination would affect thresholds for the three parameters. When 2009/2010 data were included, there was a considerable increase in threshold values with proportion ILI (**Fig. 3**). For the other two parameters, thresholds remained largely unaffected. The proposed thresholds performed similarly when applied to surveillance data from week 18 of 2014 that were not included in establishing the thresholds (**Table 2** and **Fig. 2**).

## Discussion

In the present study based on the WHO method for establishing seasonal and alert influenza thresholds, we explored a range of thresholds for three readily available parameters and established practical influenza thresholds for Cambodia. Based on consensus among national and international stakeholders, four thresholds were established to mark the start of the season and low, moderate, high and extraordinary levels of influenza activity.

When comparing the usefulness of the different parameters to monitor influenza activity, we found that the syndromic proportion ILI parameter lacked seasonality. However, proportion positive and composite, parameters that incorporate laboratory data, generally exhibited a single distinct season each year in Cambodia. Furthermore, the composite parameter using the two-consecutive-week rule to indicate season onset had no false signals during the study period.

Sensitivity analysis of thresholds with inclusion of 2009 data showed that only the proportion ILI parameter was largely affected. The considerable increase in proportion ILI during the 2009 pandemic may have been due to greater awareness resulting in more patients seeking health care for ILI and/or more clinicians categorizing patients as ILI rather than a true increase in incidence. In fact, influenza A(H1N1)pdm09 virus accounted for only one-third of all influenza subtypes detected in Cambodia during the pandemic (data not shown). These findings further confirmed the robustness of parameters and thresholds that incorporate laboratory information.

The composite variable appeared particularly useful, likely due to higher specificity for a true increase in influenza cases by accounting for both syndromic activity and laboratory positivity; for instance, it accounts for situations where there is high proportion ILI but low proportion positive as in the 2009 pandemic. Similarly, when there is high proportion positive but low proportion ILI, the composite approach would be more conservative than using proportion positive alone and would reduce false positive declarations. Additionally, in settings where the number of samples for laboratory testing is limited or small resulting in high fluctuations in proportion positive, accounting for syndromic data may be useful.

Finally, the five categories of intensity proposed here can be applied to both seasonal influenza and potential pandemic influenza within the PISA framework. (*18*) The composite approach may be especially useful for pandemic influenza assessment by accounting for a potential increase in awareness, health-care access and/or reporting. As Cambodia is one of several countries affected by human infections with avian influenza, its pandemic preparedness is especially important both domestically and globally. (*20*)

Our study has several limitations. First, the assessment was limited to approximately five to 10 laboratory samples per site per week, and reporting varied between six and eight sentinel sites during the study period. Nevertheless, the quantity and distribution of the data were sufficient to describe seasonality and establish thresholds. Next, the sentinel surveillance system covers public hospitals and health centres but not private clinics, and therefore may not be representative of the overall Cambodian population. A special study such as a health-care utilization survey is an important next step to better understand the burden of influenza nationwide. Finally, data from paediatric and non-paediatric sites were combined to establish thresholds due to sample size limitations, although paediatric sites generally had higher values for proportion positive. However, the yearly trends were similar between the two site types (data not presented). Regardless, to assess influenza activity, thresholds should be interpreted with other information such as subtypes and other parameters.

Our findings have practical public health significance. Once parameters, thresholds and categorizations are determined, it is possible to implement specific public health actions, (*21*) such as risk communication that could be triggered from crossing a threshold. In Cambodia, knowledge regarding influenza is still scarce among health workers and the general public and information regarding seasonality is just emerging. Therefore, as a first step based on these findings, the Ministry of Health plans to raise awareness among the medical community and the public regarding (1) general knowledge of influenza and its seasonality, (2) preventive measures such as respiratory and hand hygiene, and (3) prevention of antimicrobial misuse. We consider channels such as press releases, the Internet, posters and the National Respiratory Disease and Influenza Bulletin to convey these messages. In the long-term, the seasonal threshold will be helpful for vaccination timing; (*10*, *11*, *21*) continuous re-evaluation of vaccination timing will be necessary as the timing of season onset has been observed to vary in the tropics.

Although one country’s experience cannot be generalized, our findings provide novel insights with global implications, specifically for countries in the tropics and subtropics. First, ILI syndromic surveillance may not be an appropriate parameter for influenza activity in the tropics and subtropics. This contrasts to what is known for ILI data that are routinely used in temperate regions such as Europe, the United States of America and Australia as a proxy to monitor influenza activity. (*14*-*16*, *19*) Lack of apparent ILI seasonality could be unique in the tropics and subtropics with various pathogens circulating year-round that cause acute respiratory illnesses. (*8*-*11*, *21*-*32*) Instead, use of proportion positive and composite approaches may be suggested given recent studies with laboratory information indicating that most countries, including non-temperate countries, exhibit distinct seasonal patterns. (*8*-*11*, *21*-*32*) Our findings regarding the usefulness of the composite variable agree with those from a temperate region in Australia (*19*) and highlight the importance of using multiple sources of information to guide assessment. Considering similar surveillance systems in Cambodia and those in other tropical and subtropical countries, (*21*-*28*) our approach may be adapted to fit each country’s context. For Cambodia, eight sentinel sites with approximately 35 samples per week nationwide were enough to describe influenza activity. Furthermore, there are several key observations in influenza activity that are unique to the tropics and subtropics: (*9*-*11*, *27*, *29*) (1) annual timing of season onset and peak vary considerably, (2) season onset appears more gradual, and (3) magnitude of influenza season is not as distinct from off-season. These were also observed in the present study (**Fig. 2**). Such characteristics make it especially meaningful to set explicit thresholds based on appropriate parameters to support routine public health communications and allocate resources effectively and efficiently. (*21*) In addition to the leadership of respective ministries of health, global efforts by WHO, the Centers for Disease Control and Prevention and other organizations have supported the establishment of national influenza surveillance systems in many resource-limited countries in non-temperate climates. (*10*, *11*, *21*-*34*) We believe it is time to maximize utilization of influenza surveillance data for routine actions for domestic and global public health assessment and response.

In summary, distinct seasonality of influenza activity in Cambodia was observed using two parameters that incorporate laboratory information, allowing for the establishment of thresholds and transmission intensity categories. The composite variable that accounts for syndromic and laboratory data was the most specific in declaring season onset and the most useful in monitoring intensity. This categorization can assess not only seasonal influenza but also potential pandemic influenza, contributing to the country’s pandemic preparedness. These findings have important implications for countries in the tropics, subtropics and in resource-limited settings.
